# Metastatic Breast Cancer Presenting as Acute Appendicitis: A Case Report and Literature Review

**DOI:** 10.7759/cureus.59682

**Published:** 2024-05-05

**Authors:** Meshael S Albahli, Norah I Alabdulaaly, Ahmed A Alzahrani, Ashwag H AlHarbi, Ahmad M Alshihri, Hussam A Alharbi, Feras Alsannaa

**Affiliations:** 1 General Surgery, Prince Sultan Military Medical City, Riyadh, SAU; 2 General Surgery, Alkharj Armed Forces Hospital, Riyadh, SAU

**Keywords:** atypical presentation, cancer, metastasis, breast cancer, acute appendicitis

## Abstract

Breast cancer is considered one of the most common cancers worldwide. The most common sites for breast cancer to metastasize are the lymph nodes, bones, lungs, brain, and liver. Involvement of the gastrointestinal tract is uncommon, and metastasis to the appendix is rare. We report a case involving a 43-year-old woman with no previous history of malignancy who presented with acute appendicitis and underwent laparoscopic appendectomy, with the final histopathological assessment revealing metastatic breast cancer.

## Introduction

Breast cancer is considered one of the most common types of cancer worldwide [1]. The breast cancer five-year survival rate reached 92% in 2013-2017. The five-year survival rate has improved significantly when compared to the rate in 1988-1992 which was 76% [2]. The main reason behind this improvement is the screening programs, which led to the early detection of the disease as well as the improvement in the surgical, radiological, and systemic treatment methods [3]. The most common sites of metastasis are the lymph nodes, lungs, bones, brain, and liver. The involvement of the gastrointestinal tract can happen, but it is uncommon, and in particular, metastasis to the appendix is rare [4-6]. The commonest presentation for these patients is acute appendicitis, for which they eventually undergo an appendectomy. The prognosis and survival are not affected and are mainly dependent on the subsequent systemic treatment [7].

## Case presentation

A 43-year-old female presented to the emergency department (ED) with a one-day history of right lower quadrant (RLQ) abdominal pain. The pain was colicky, gradually appearing in the paraumbilical area and then shifting to the RLQ. The pain was associated with multiple episodes of vomiting, had no aggravating factor, and was relieved partially by analgesia. The patient denied any history of fever, chills, urinary symptoms, constipation, or diarrhea. Her past medical history was significant for type 2 diabetes mellitus and hypothyroidism; however, her surgical history was not significant.

On presentation to the ED, the patient had a heart rate of 108 beats per minute and a body temperature of 37.2°C. However, the other vital signs were stable. She was lying on the bed comfortably, in mild pain. Her abdominal examination showed no previous surgical scars and a soft but tender right lilac fossa, positive rebound tenderness, and a negative Rovsing sign. A breast examination showed a hard, ill-defined breast mass in the left breast with palpable axillary lymph nodes.

**Figure 1:**
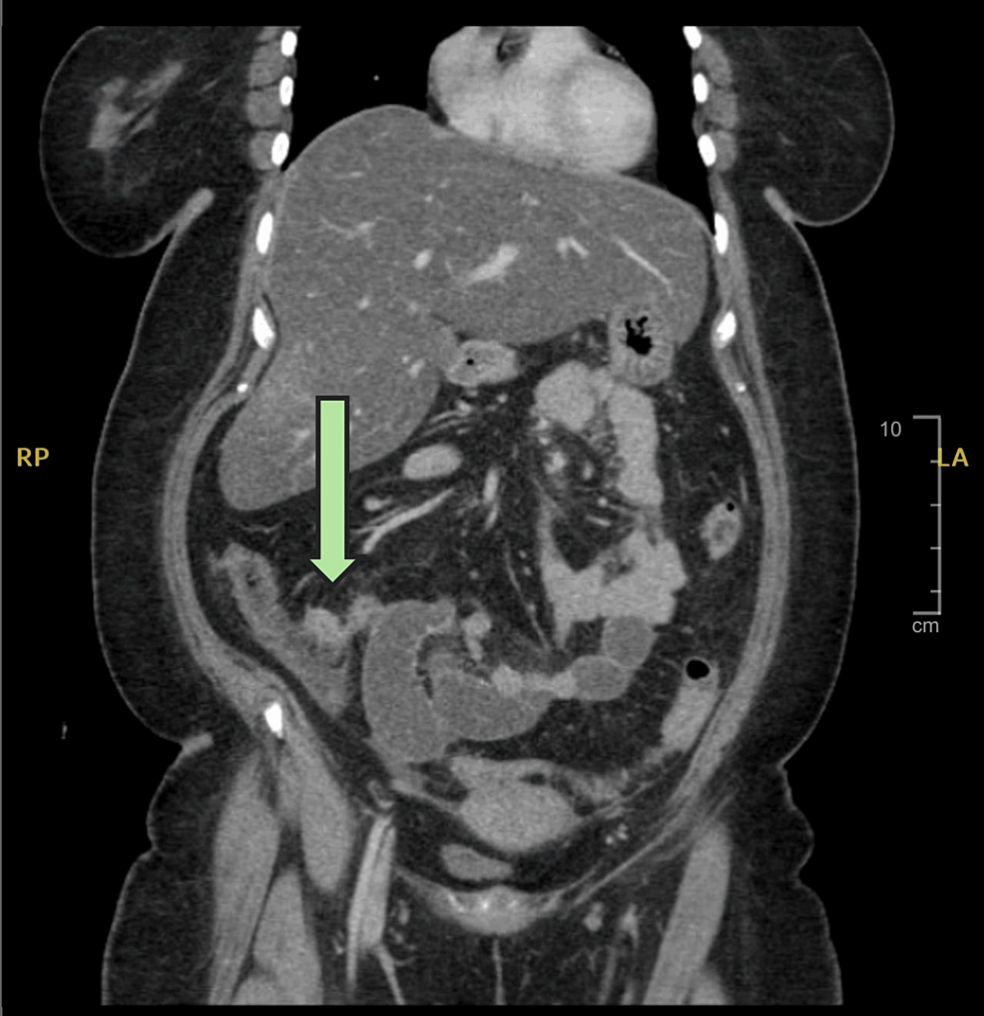
CT abdomen with contrast CT: Computed tomography The arrow indicates the enhanced structure with wall discontinuity.

The patient underwent a full blood workup, including a complete blood count and electrolyte, renal, and liver function profiles. The tests showed that the white blood cell count had increased to 16.33 × 109/L, while the neutrophil count had increased to 13.53 × 109/L. However, the results of other laboratory examinations were unremarkable. A computed tomography (CT) scan with intravenous contrast administration showed that the appendix was not visible. However, an enhancing structure with wall discontinuity was observed at the base of the cecum, indicating a ruptured appendix, and low-grade small bowel obstruction was also observed. In addition, the scans showed severe hepatic steatosis and multiple breast nodules with widespread heterogenous sclerotic and lytic bony lesions (Figure 1).

**Figure 2:**
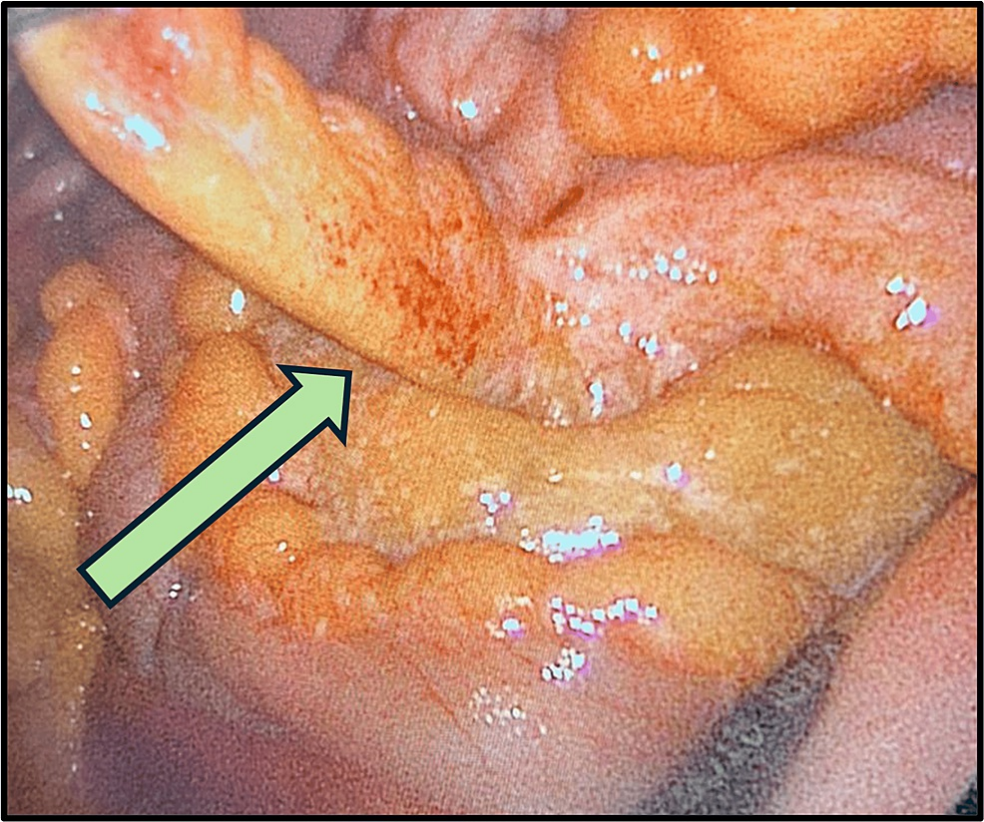
Laparoscopic view showing an inflamed appendix The arrow is pointing to the inflamed appendix.

**Figure 3:**
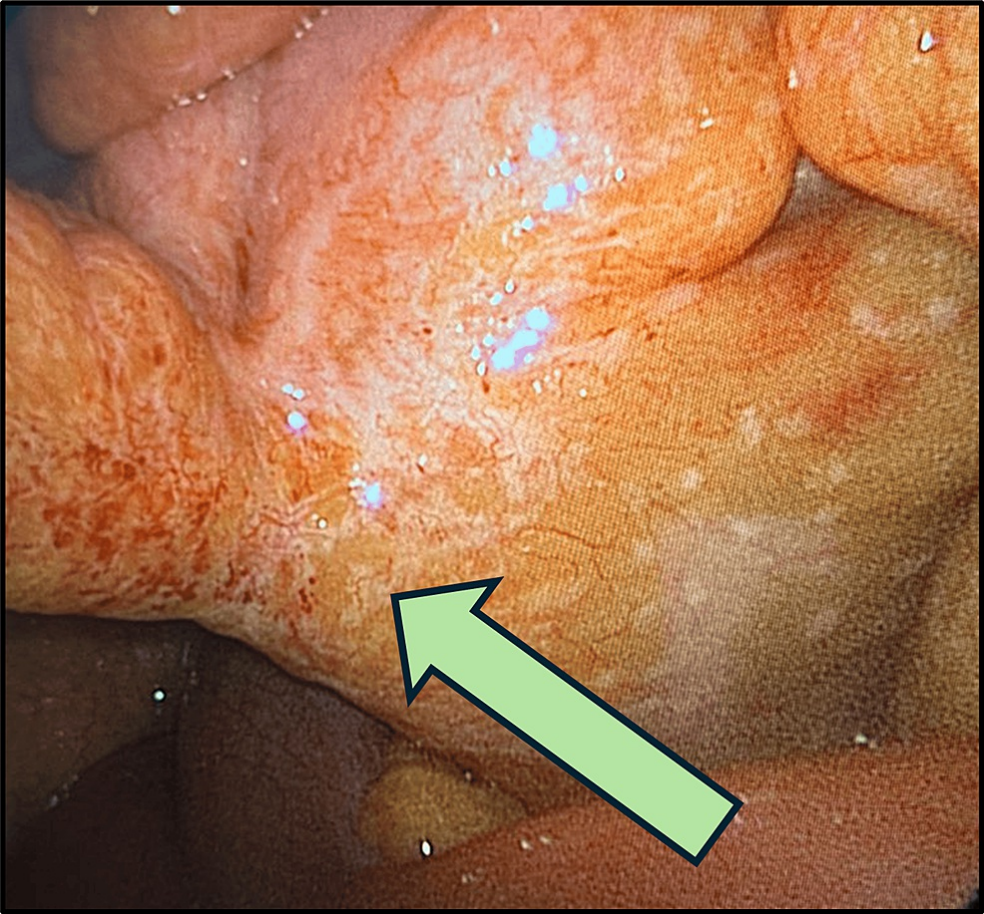
Laparoscopic view showing a healthy base of the appendix The arrow is pointing to a healthy base of the appendix.

**Figure 4:**
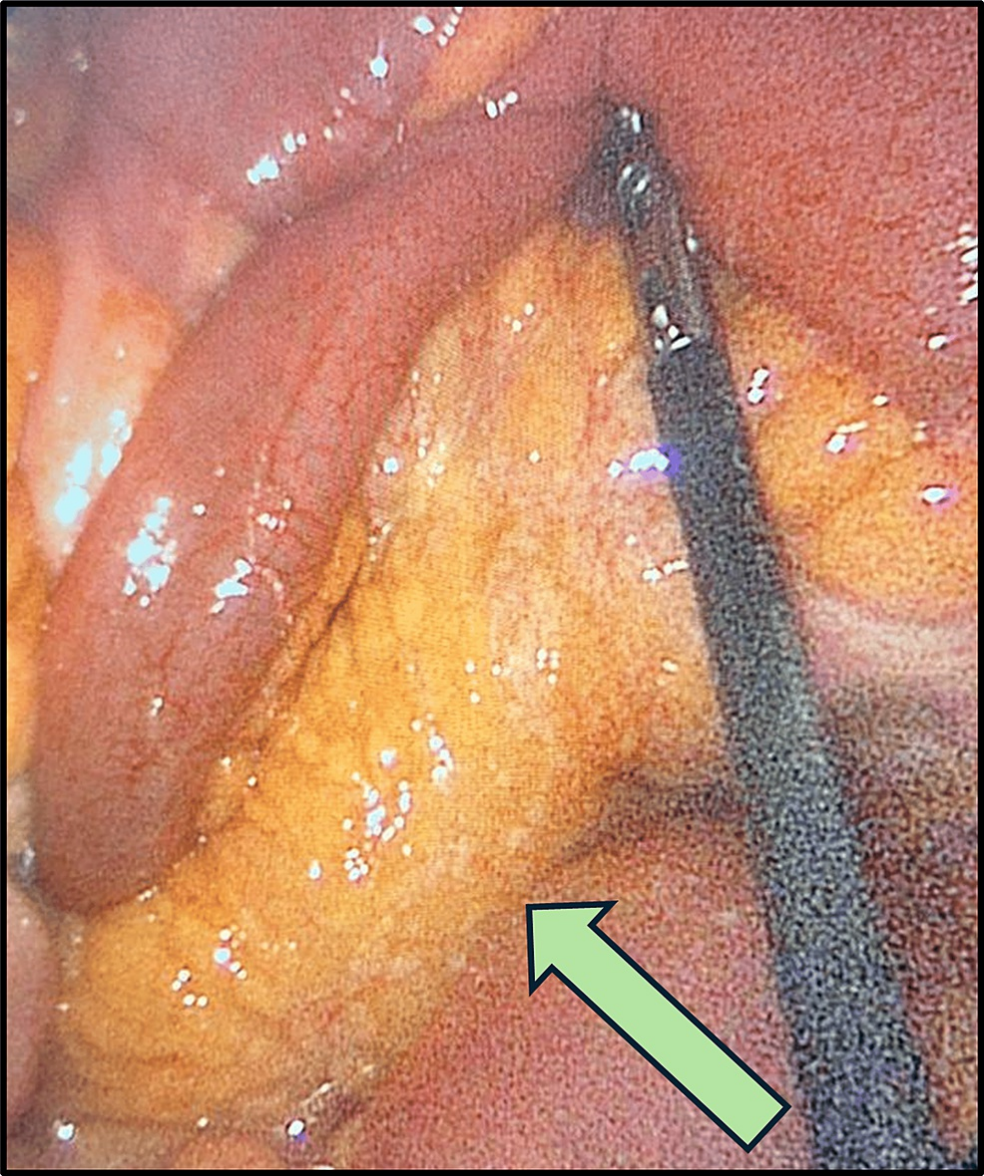
Laparoscopic view showing omental caking The arrow is pointing to the omental caking.

**Figure 5:**
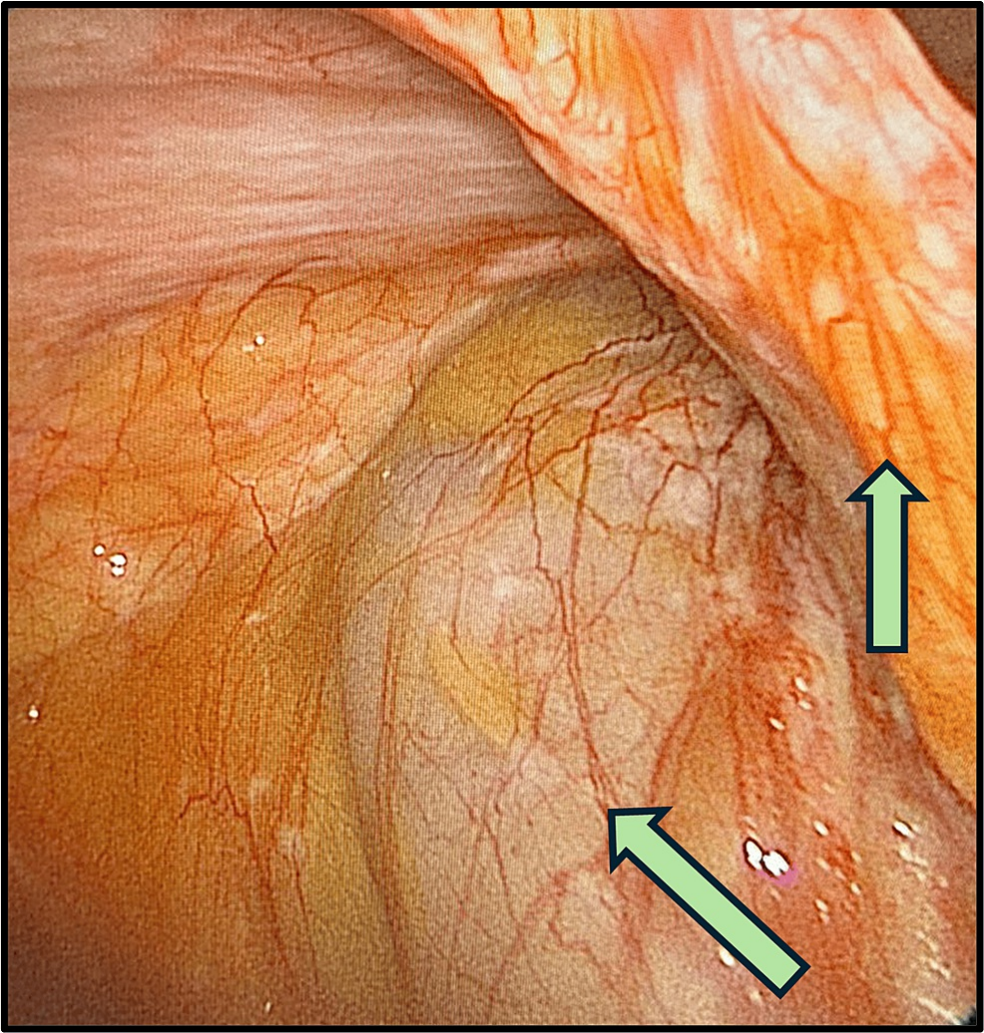
Laparoscopic view showing peritoneal deposits The arrow is pointing to the peritoneal deposits.

The patient was admitted and planned for laparoscopic exploration with a possible appendectomy. The operative findings were an inflamed, non-ruptured appendix (Figure 2), a healthy base (Figure 3), omental caking (Figure 4), and peritoneal deposits (Figure 5), while the ovaries were normal. The base of the appendix was collected with an endo-GIA stapler device. The appendix was sent for histopathological assessment, and the free pelvic fluid was obtained for cytological analysis and assessment of acid-fast bacilli, other bacteria, and fungi.

The patient was shifted to the intensive care unit for observation for one day and then shifted to the ward in good condition.

Based on the abdominal CT and operative findings, a chest CT was performed, which showed bilateral small breast nodules, enhanced borderline left axillary lymph nodes, and diffuse sclerotic bony lesions indicating metastasis. The right upper lobe showed a 3-mm nodule. A mammogram/ultrasound-guided biopsy performed during the same admission showed left breast findings indicating multifocal breast cancer and suspected axillary lymph node metastasis. Moreover, a bone scan showed patchy radiotracer distribution in the anterior right ribs and posterior left ribs, which was associated with heterogeneous tracer uptake in the dorsal and lumbar vertebrae. After completing the investigation in collaboration with the breast and endocrine teams, the patient was discharged on day three with follow-up at the breast and endocrine surgery clinic.

When the patient presented to the clinic two weeks later, her cytological findings showed reactivity for mesothelial cells against a background of inflammatory cells. Cultures for acid-fast bacilli, other bacteria, and fungi showed negative results, and the findings of body fluid analysis were unremarkable. However, the histopathological findings for the appendix showed a metastatic carcinoma consistent with a breast origin, and the patient’s breast biopsy showed a grade 2 invasive lobular carcinoma. Immunohistochemical studies for breast biomarkers showed positive results for estrogen and progesterone receptors and negative results for human epidermal growth factor receptor 2 (HER-2/neu).

A tumor board meeting recommended a positron emission tomography (PET) scan for the final management plan to be decided. The PET scan showed diffuse axial sclerotic lesions with heterogeneous FDG uptake, most of which involved the L3 and T7 regions and the posterior column of the left acetabulum. Based on the positive results for metastasis in the PET scan, the patient was referred to the oncology department for medical treatment. A full oncological workup revealed high levels of carcinoembryonic antigen (6) and carbohydrate antigen 15-3 (33), and chemotherapy was planned.

## Discussion

Bone is the most common site of breast cancer metastasis [8]. Metastases to the gastrointestinal tract of breast carcinoma are rare and account for less than 15% of breast carcinoma metastases, which makes the diagnosis challenging and leads to difficulties in management. In patients showing gastrointestinal tract metastases, the stomach, small bowel, and large bowel are most commonly the parts to be affected. The metastasis to the appendix is a rare finding that can happen over a long period between the diagnosis of the primary carcinoma and the metastases [6,9-11]. The spread of breast cancer metastases is mediated by two major factors: first, estrogen receptor status, in which estrogen receptor-negative tumors show a greater likelihood of metastases to the gastrointestinal tract. Second, the pathology of the cancer, in which lobular carcinomas show a greater likelihood of metastasis to the gastrointestinal tract than the others [12,13].

According to the 1998 Connor et al. retrospective study of the histopathological findings from appendicectomies, which concluded that cancer metastases in the appendix are rare, of the 7,970 results analyzed, only 0.9% represented tumors, and of all that, carcinoid tumors were the most common. Only 15% of all tumors were due to secondary malignancies, and from that, most of the cases were colorectal metastatic cancers in origin [14].

The hallmark histological finding of appendicular metastatic cancer is gradual serosal involvement with a usually intact mucosal layer [15]. The incidence of benign disorders of the appendix, like acute appendicitis and primary tumors of the appendix, is much higher than that of metastatic tumors in patients with a known history of malignancy, which makes the clinical diagnosis of such conditions challenging [7]. Patients who present with metastasis in the gastrointestinal tract frequently will have an extended disease in other organs [9]. Due to radiation or chemotherapy for metastatic disease, the signs and symptoms of acute appendicitis are usually not typical, which leads to delay and uncertainty in the diagnosis [16]. Radiological investigations have an important role in the diagnosis, with CT being the gold standard [17]. PET scanning can help diagnose patients with advanced-stage cancer who do not present with typical abdominal pain. However, it is difficult to distinguish between non-tumoral perforated appendicitis and perforated appendicular tumors [18].

Due to the improvement of multidisciplinary team approaches to cancer patients, which resulted in extending cancer patients' survival times, an increased incidence of uncommon breast cancer metastases was observed. But it may also be related to the progression of the disease, as it is resistant to conventional therapies [19]. The median survival is poor after the detection of distant gastrointestinal metastases [7]. For such cases, the only factor noticed to prolong survival is systemic therapy [6,7,10]. Acute appendicitis secondary to metastatic breast cancer metastases can be treated with a simple appendectomy. There are no previous reports to analyze if right hemicolectomy can lead to better oncological outcomes compared to appendectomy only. Some authors recommend prophylactic appendectomy in patients who will require abdominal surgery for other reasons, but this approach is not supported by evidence [20].

## Conclusions

Breast cancer metastases to the gastrointestinal tract are rare. Furthermore, appendicitis caused by metastatic breast cancer is considered to be very rare. Due to the improvement in systemic therapy, which leads to increased survival in patients with advanced-stage breast cancer, uncommon locations for distal metastases have been noticed more frequently. The diagnosis of such unusual sites of metastasis is difficult and requires a combination of clinical, laboratory, and radiological findings to confirm the diagnosis. Nevertheless, involvement of the gastrointestinal tract can be predicted and observed, especially in patients with a history of advanced-stage (stage IV) breast cancer. Although appendicitis is mainly considered to be an inflammatory process, in patients with a history of advanced breast cancer, oncological etiologies must be considered to identify the appropriate treatment approach. Early intervention in such cases helps prevent severe complications such as sepsis. Although such management does not affect the survival of the primary cancer, multidisciplinary management can lead to better outcomes.
